# Using a hepatitis B surveillance system evaluation in Fujian, Hainan, and Gansu provinces to improve data quality and assess program effectiveness, China, 2015

**DOI:** 10.1186/s12879-020-05265-3

**Published:** 2020-07-25

**Authors:** Hui Zheng, Alexander J. Millman, Jeanette J. Rainey, Fuzhen Wang, Rui Zhang, Hong Chen, Zundong Yin, Huaqing Wang, Guomin Zhang

**Affiliations:** 1 National Immunization Program, Chinese Center for Disease Control and Prevention, Beijing, China; 2 Division of Viral Hepatitis, United States Centers for Disease Control and Prevention, Atlanta, GA USA; 3 Division of Global Health Protection, United States, Centers for Disease Control and Prevention, Atlanta, GA USA; 4 Emerging Infections Program, Chinese Center of Disease Control and Prevention, Beijing, China

**Keywords:** Hepatitis B; acute hepatitis infection, Chronic hepatitis infection, Surveillance, Case-reporting

## Abstract

**Background:**

Monitoring hepatitis B surveillance data is important for evaluating progress towards global hepatitis B elimination goals. Accurate classification of acute and chronic hepatitis infections is essential for assessing program effectiveness.

**Methods:**

We evaluated hepatitis B case-reporting at six hospitals in Fujian, Hainan and Gansu provinces in 2015 to assess the accuracy of case classification. We linked National Notifiable Disease Reporting System (NNDRS) HBV case-reports with hospital information systems and extracted information on age, gender, admission ward and viral hepatitis diagnosis from medical records. To assess accuracy, we compared NNDRS reported case-classifications with the national HBV case definitions. Multivariable logistic regression was used to identify factors associated with misclassification.

**Results:**

Of the 1420 HBV cases reported to NNDRS, 23 (6.5%) of the 352 acute reports and 648 (60.7%) of the 1068 chronic reports were correctly classified. Of the remaining, 318 (22.4%) were misclassified and 431 (30.4%) could not be classified due to the lack of supporting information. Based on the multivariable analysis, HBV cases reported from Hainan (aOR = 1.8; 95% *CI*: 1.3–2.4) and Gansu (aOR = 12.7; 95% *CI*: 7.7–20.1) along with reports from grade 2 hospitals (aOR = 1.6; 95% *CI*:1.2–2.2) and those from non-HBV related departments (aOR = 5.3; 95% *CI*: 4.1–7.0) were independently associated with being ‘misclassified’ in NNDRS.

**Conclusions:**

We identified discrepancies in the accuracy of HBV case-reporting in the project hospitals. Onsite training on the use of anti-HBc IgM testing as well as on HBV case definitions and reporting procedures are needed to accurately assess program effectiveness and ensure case-patients are referred to appropriate treatment and care. Routine surveillance evaluations such as this can be useful for improving data quality and monitoring program effectiveness.

## Background

Globally, 257 million persons have chronic hepatitis B virus infections (HBV) and nearly 900,000 HBV-related deaths occur annually [1]. To address this disease burden, the World Health Organization (WHO) outlined a new strategy for viral hepatitis elimination, focusing on a 90% reduction of new chronic viral hepatitis B cases by 2030 [2]. China has reduced HBV transmission in persons born after 1992 through the successful implementation of a HBV vaccination program [3, 4]. By 2010, more than 98% of children were completing the three-dose hepatitis B vaccination series each year [5]. However, an estimated 90 million individuals of whom the majority are older than 30 years of age [6], are HBV surface antigen positive (HBsAg+) and at risk of developing cirrhosis and liver cancer [7, 8]. Persons who are HBsAg+ can transmit HBV to susceptible persons.

Approximately 10% of the population in China was identified as HBsAg+ in a 1992 sero-survey [9]. To monitor changes in the prevalence of HBsAg+, the National Health Commission (NHC) (formerly the National Health and Family Planning Commission) implemented a policy requiring hospital staff to report all newly identified HBsAg+ case-patients to the National Notifiable Disease Reporting System (NNDRS). NNDRS is a passive web-based surveillance system that relies on clinicians to report HBV infections as either acute, chronic or non-classifiable HBV infections, based on the case definitions outlined in the national HBV reporting guidelines [10]. The system is available in all hospitals in China and can be used to monitor the accuracy and occurrence of acute and chronic HBV case reports. Passive surveillance is generally less costly to implement and maintain than active surveillance. Relying on clinicians for case reporting, however, can negatively affect the accuracy of the surveillance data, particularly if interpretations of the case definitions and diagnostic criteria are highly variable [11–13]. Previous evaluations of acute HBV case-reporting to NNDRS in Yunnan, Shanghai, Tianjin, and Qinghai, for example, indicated that only 4–37% of acute cases were reported correctly, according to the national case definitions [14–18]. These findings can affect the validity of HBV incidence estimates, the timely identification of HBV outbreaks, and the ability to appropriately target HBV prevention and control interventions.

In this project, we evaluated the accuracy of HBV surveillance data reported to NNDRS from hospitals in three geographically and demographically diverse provinces and identified factors that may affect the accuracy of these reports. We anticipate that the findings from this project can be used to strengthen China’s HBV surveillance program to monitor the occurrence of acute and chronic infections and to assist it in achieving its global hepatitis B elimination goals [2]. When combined with more resource intensive sero-surveys [19, 20], the methods described in this paper could also be adapted and implemented in other high HBV burden countries as a supplemental tool for monitoring hepatitis B program effectiveness.

## Methods

### Project site selection

**Fig. 1:**
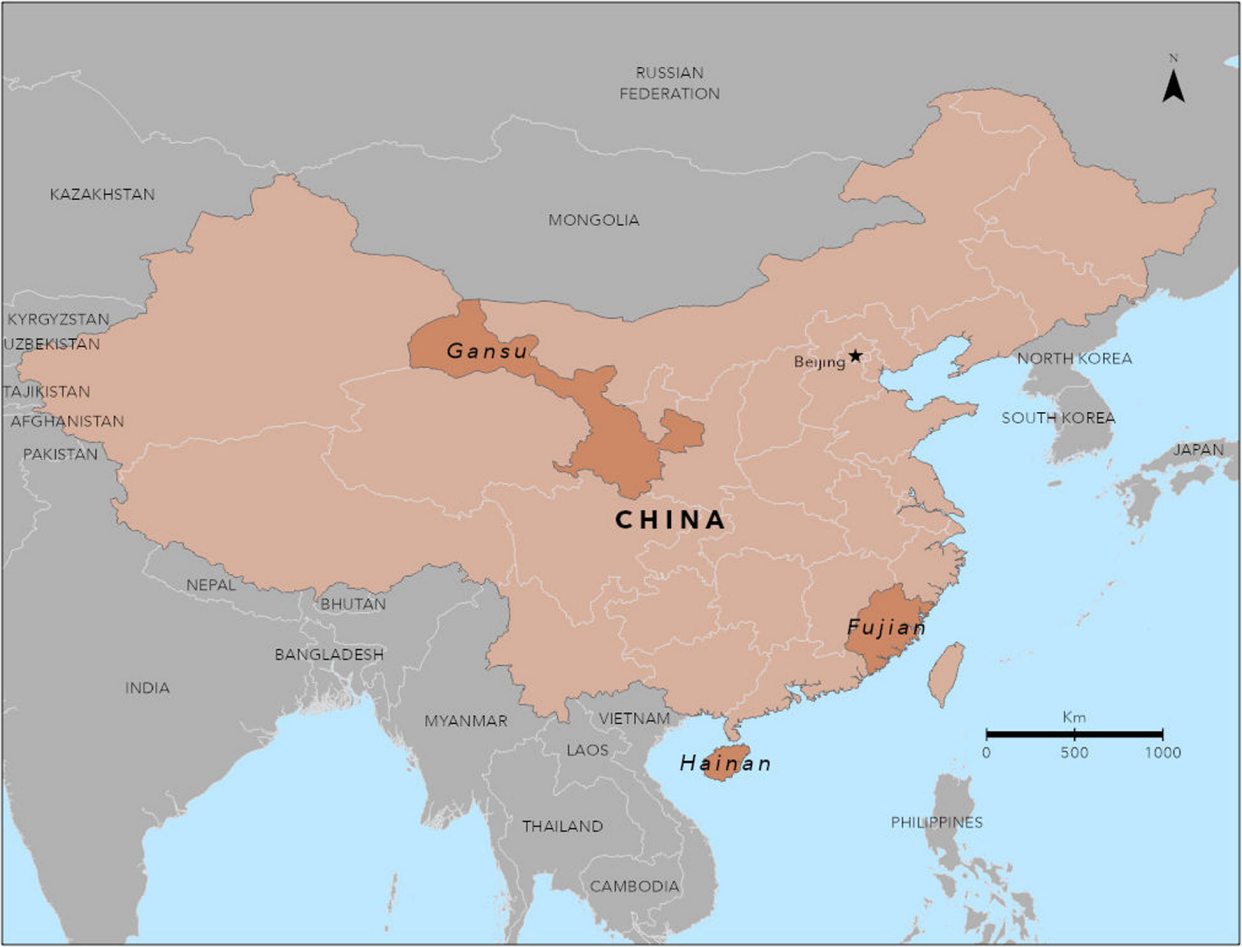
Location of Fujian, Hainan, and Gansu Provinces participating in the hepatitis B surveillance project, China 2015

We evaluated HBV case-reporting in Fujian, Hainan, and Gansu Provinces (Fig. 1). Fujian is located on the eastern coast and has a population of 37 million; Gansu is located in the west and has a population of 26 million; and Hainan, is the smallest province and an island, with a population of approximately 9 million in 2010 [21]. The estimated population prevalence of HBsAg+ is 4.4% in Gansu, 11.9% in Hainan, and 15.5% in Fujian [22].

In China, NHC rates hospitals from grade 1 to 3 based on the level of service availability. Grade 3 hospitals offer the highest comprehensive level of service. In each province, we selected one grade 2 and one grade 3 hospitals for a total of six project hospitals using the following criteria: 1) the hospital had an advanced laboratory information system (LIS) with access to HBsAg test results; 2) the hospital had an electronic hospital information system (HIS) that can link the LIS to the inpatient medical record number; and 3) in 2015, the hospital reported a greater number of hepatitis B cases compared to the hospital-based provincial mean. Additionally, administrators at each hospital agreed to participate in the project. Because more than 90% of hepatitis B cases are reported from grade 2 and 3 hospitals, we elected not to include other types of health care facilities in this project.

### Case inclusion criteria and data collection

We included hepatitis B cases from the six participating hospitals that were reported to NNDRS from 1 January to 31 December 2015 and had a LIS record indicating HBsAg+ test result for the same time-period. We downloaded all HBV cases that were reported to NNDRS from the six hospitals and linked these case reports to LIS data by name, gender, and birthdate. NNDRS records that could not be linked to LIS data and outpatients without record numbers were excluded from the analysis. We developed a standard abstraction form to collect medical record data from the six project hospitals for the reported HBV cases that were successfully linked to LIS. Staff from the Chinese Center for Disease Control and Prevention (China CDC) and the six hospitals used the form to collect the following information: reason for admission, admission ward (HBV-related wards included internal medicine, infectious disease, gastroenterology, and liver disease; non-HBV-related wards included surgery, pediatrics, obstetrics and gynecology, pulmonology, endocrinology, nephrology, orthopedics, neurosurgery, and traditional Chinese medicine), discharge diagnoses, clinical information including signs and symptoms of viral hepatitis, liver function tests, HBV DNA viral load, and hepatitis B sero-markers including HBV core antibody IgM (anti-HBc IgM), IgM antibody for hepatitis A virus (anti-HAV IgM) and hepatitis E virus (anti-HEV IgM), and hepatitis C virus antibody (anti-HCV). We obtained information on a patient’s classification as an acute or chronic hepatitis B case from the hospital’s information system.

### Data management and analysis

We double-entered and verified data abstracted from the medical record in EpiData (version 3.1, Denmark). The resulting EpiData database was merged with the NNDRS-LIS case data using the patient medical record number and imported into SPSS (version 23, IBM, New York, USA) for analysis. We generated descriptive statistics on HBV cases by province, hospital grade, ward type, and patient characteristics. We compared the classification of hepatitis cases reported to NNDRS with the national HBV case definitions.

According to the national case definitions, hepatitis B patients should be classified as follows:

1. *HBV carriers*: HBsAg-positive patient with no signs or symptoms of liver disease and normal alanine aminotransferase (ALT) levels (≤40 IU/mL).
2. *Acute HBV*: HBsAg-positive patient, who has signs or symptoms of liver disease or abnormal ALT levels (> 40 IU/mL) without chronic inflammatory changes reported on abdominal ultrasound and negative hepatitis A and hepatitis E serologies, and who has clear evidence of HBV infection for less than 6 months or anti-HBc IgM positive.
3. *Chronic HBV*: HBsAg-positive patient, who has signs or symptoms of chronic liver disease or abnormal ALT levels (> 40 IU/mL), and with at least one of the following: previous history of HBV infection ≥6 months before, chronic inflammatory changes reported on abdominal ultrasound, or anti-HBc IgM negative.
4. *Cirrhosis*: HBsAg-positive patient with liver cirrhosis as reported via abdominal ultrasound, computerized tomography (CT), or magnetic resonance imaging (MRI).
5. *Hepatocellular carcinoma (HCC)*: patient with liver lesion(s) that are suggestive of HCC reported via abdominal ultrasound, CT, or MRI together with alpha fetoprotein (AFP) > 400 μg/mL.
6. *Non-classifiable HBV*: HBsAg-positive patient with only signs and symptoms of liver disease and no additional information, or an HBsAg-positive patient with insufficient information for classification.

We generated descriptive statistics on the clinical characteristics and laboratory test results from HBV patients who were included in the final database. Patients who were reported to NNDRS as having acute HBV infection were considered to be correctly classified if they met the case definition for acute HBV based on the medical record review. Similarly, patients reported to NNDRS as having chronic HBV were considered to be correctly classified if they met the case definition of chronic HBV, cirrhosis, or HCC, based on the medical record review. The remaining patients were considered misclassified, or unable to be classified because of missing laboratory data. We calculated the percentage of patients who were correctly and incorrectly classified by province, hospital grade, gender, age, ward type, and primary discharge diagnosis (HBV or other). We stratified this analysis by NNDRS reporting status (i.e., reported as an acute or chronic HBV patient). In this bivariate analysis, all hospital and patient characteristics, except for province (three categories) and age (four categories), were dichotomized. An alpha level of 0.05 was applied to assess statistical significance.

Using classification status—NDRSS case-reports that were correctly classified and case-reports that were misclassified (including unable to classify)—as our binary dependent variable, we developed a logistic regression model to estimate the crude association of each of the above characteristics (e.g., province, hospital grade, and ward type) with an incorrect HBV case classification. Characteristics that were statistically significant in the crude analysis were included in our multivariable logistic regression model. Unadjusted and adjusted odds ratios (OR) and 95% confidence intervals (*CI*) were calculated. Finally, using available medical record information, we described the characteristics of HBV case-reports that were misclassified as acute and chronic in NNDRS.

### Ethics approval

The China CDC Ethics Review Committee approved the project as a program evaluation. The United States Centers for Disease Control and Prevention approved the project as a routine surveillance activity. We maintained all project-related data on a secure and password-protected computer.

## Results

**Fig. 2:**
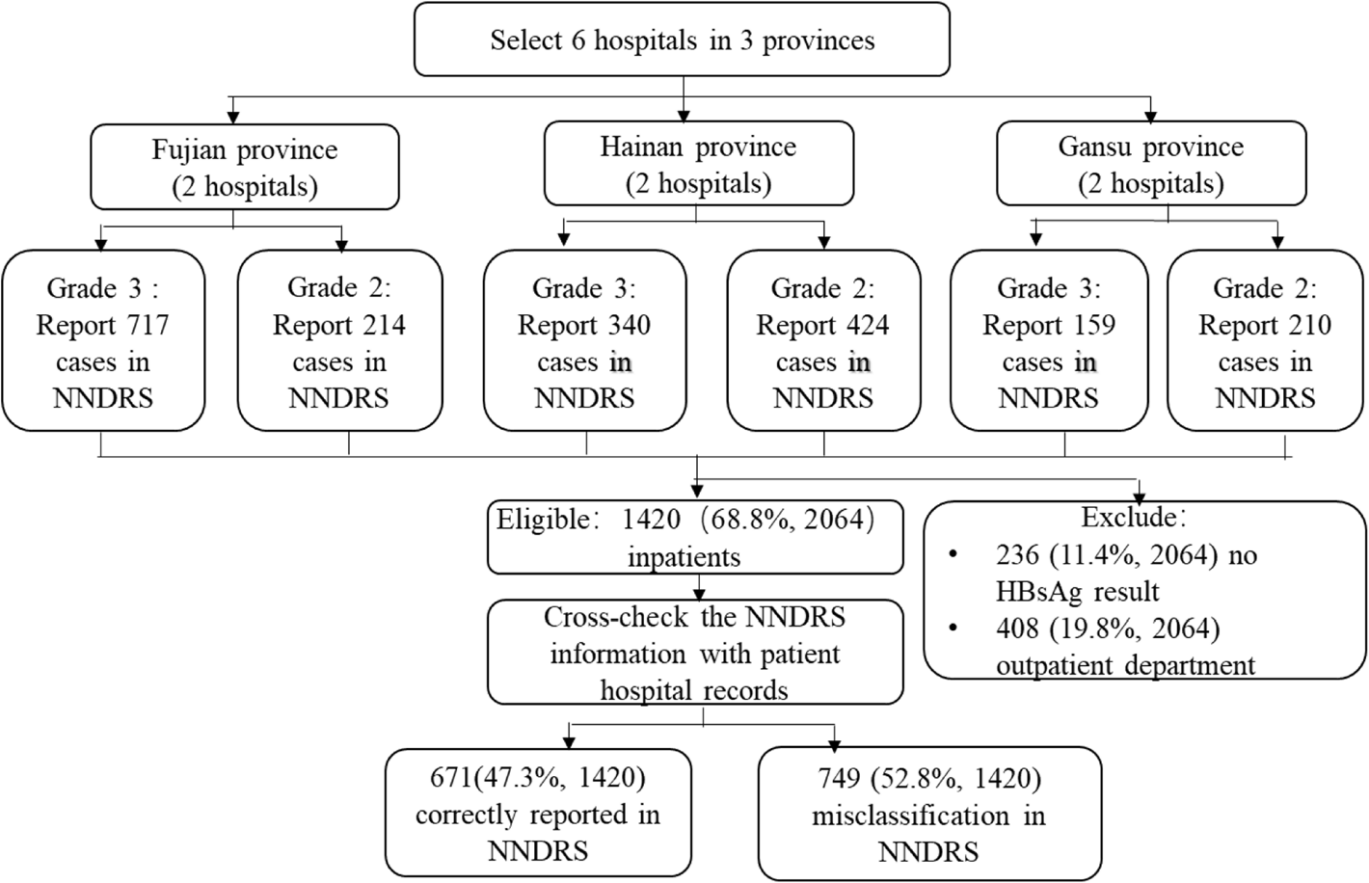
Identification of eligible cases for review from six project hospitals in Fujian, Hainan, Gansu Provinces, China, 2015

From 1 January–31 December 2015, 2064 HBV patients were reported to NNDRS by the six participating hospitals. Of these, 644 (31.2%) were excluded from the analysis: 236 did not have HBsAg+ test results in LIS and 408 were outpatients (Fig. 2). Among the remaining 1420 patients, 881 (62.0%) were from Fujian Province, 267 (18.8%) were from Hainan, and 272 (19.2%) were from Gansu. Most patients (*n* = 1014, 71.4%) were reported from grade 3 hospitals, were male (*n* = 991, 69.8%), and were ≥ 40 years of age (*n* = 873, 61.5%).

The percentage of cases reported to NNDRS as chronic HBV was 12.5% (34/272) in Gansu Province, 61.8% (165/267) in Hainan, and 98.6% (869/881) in Fujian. The percentage of cases reported to NNDRS as acute HBV was 87.5% (238/272) in Gansu Province, 38.2% (102/267) in Hainan, and 1.4% (12/881) in Fujian. Across all project hospitals, the majority (57.7%) of HBV cases were reported from HBV-related wards (Table 1). A larger percentage of patients from HBV-related departments were assessed for signs and symptoms of HBV infection and underwent an abdominal ultrasound or testing for HBV DNA, hepatitis A, C, and E compared to patients from non-HBV-related wards (data not shown, all *p*-values < 0.05). Regardless of the ward, only 1.4% of all patients underwent anti-HBc IgM testing. Of the 352 reported acute HBV patients, only 34.4, 0.3, and 2.8% of patients received testing for anti-HAV IgM, anti-HEV IgM, and anti-HBc IgM, respectively. Less than half of all HBV patients had medical record documentation of a hepatitis B diagnosis.

**Table 1:** Demographic and clinical information on hepatitis B patients reported to the National Notifiable Disease Reporting System (NNDRS) from six project hospitals in Fujian, Hainan and Gansu Provinces, China, 2015

	Fujian (***N*** = 881)		Hainan (***N*** = 267)		Gansu (***N*** = 272)		Total (***N*** = 1420)	
	N	%	n	%	N	%	N	%
**Hospital Grade**								
2	191	21.7	95	35.6	120	44.1	406	28.6
3	690	78.3	172	64.4	152	55.9	1014	71.4
**Gender**								
Male	632	71.7	189	70.8	170	62.5	991	69.8
Female	249	28.3	78	29.2	102	37.5	429	30.2
**Age group(year)**								
0–24	64	7.3	38	14.2	28	10.3	130	9.2
25–39	283	32.1	84	31.5	50	18.4	417	29.4
40–59	384	43.6	95	35.6	131	48.2	610	43.0
≥ 60	150	17.0	50	18.7	63	23.2	263	18.5
**Report in NNDRS**								
Acute hepatitis B	12	1.4	102	38.2	238	87.5	352	24.8
Chronic hepatitis B	869	98.6	165	61.8	34	12.5	1068	75.2
HBV-related departments*								
Yes	612	69.5	126	47.2	81	29.8	819	57.7
No	269	30.5	141	52.8	191	70.2	601	42.3
**Primary discharge diagnosis**								
Yes	385	43.7	91	34.1	36	13.2	512	36.1
No	496	56.3	176	65.9	236	86.8	908	63.9
**Specified acute or chronic diagnosis by clinicians**								
Yes	429	48.7	84	31.5	81	29.8	594	41.8
No	452	51.3	183	68.5	191	70.2	826	58.2

*HBV – hepatitis B virus

**Fig. 3:**
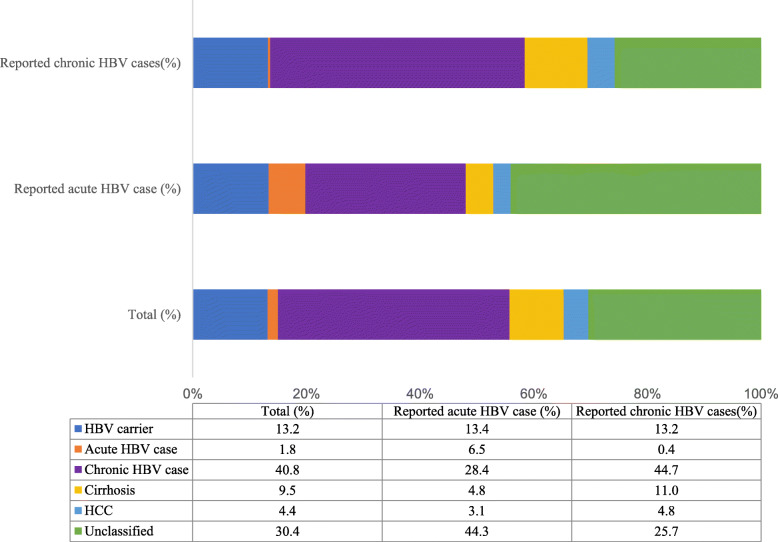
Corrected case-classification of Hepatitis B cases reported to the National Notifiable Disease Reporting System from six project hospitals based on information abstracted from patients’ medical records, Fujian, Hainan and Gansu Provinces, China, 2015

Of the 352 patients reported to NNDRS as having acute HBV infections, 23 (6.5%) were correctly classified as having acute HBV. The remaining 329 included 45 (12.8%) patients who should have been reported as HBV carriers, 100 (28.4%) as chronic HBV cases, 17 (4.8%) as cirrhotic cases, 11 (3.1%) with HCC, and 156 (44.3%) who were non-classifiable (i.e., missing diagnostic information) (Fig. 3). Of the 1068 patients who were reported to NNDRS as having a chronic infection, 648 (60.7%) were correctly classified in NNDRS at the time of the initial report. The remaining 420 included 143 (13.4%) patients who should have been reported as HBV carriers, two (0.2%) as having an acute HBV infection, and 275 (25.7%) who were considered to be non-classifiable. In our bivariate analysis of acute HBV case-reports to NNDRS, province, hospital grade, ward type (HBV or non-HBV related), and having a discharge diagnosis of HBV were statistically associated with an incorrect HBV case-classification (all *p*-values < 0.05; Table 2). Of the chronic HBV case-reports to NNDRS, gender, age, ward type, and having a discharge diagnosis of HBV were statistically associated with an incorrect case-classification (all *p*-values < 0.05). Overall, 25 (1.8%) of the reported HBV patients could be considered to have acute infections based on the available information. Of these, 19 (76%) were from Hainan Province, and the remaining five (20%) were less than 24 years of age and eligible to have received the HBV vaccination as children.

**Table 2:** Hepatitis B cases reported to the National Notifiable Disease Reporting System by original and revised* case classification from six project hospitals in Fujian, Hainan, and Gansu Provinces, China, 2015

	Cases	Acute Hepatitis B				Chronic Hepatitis B			
		Reported	Revised	% Accurate	χ^**2**^, ***p***-value	Reported	Revised	% Accurate	χ^**2**^, ***p***-value
**Province**					< 0.001				0.18
Fujian	881	12	1	8.3		869	538	61.9	
Hainan	267	102	17	16.7		165	93	56.4	
Gansu	272	238	5	2.10		34	17	50.0	
**Hospital Grade**					0.004				0.41
2	406	223	21	9.4		183	106	57.9	
3	1014	129	2	1.6		885	542	61.3	
**Gender**					1.0				< 0.001
Male	991	218	14	6.4		773	498	64.4	
Female	429	134	9	6.7		295	150	50.9	
**Age group(year)**					0.33^#^				0.007
0–24	130	45	5	11.1		85	60	70.6	
25–39	417	91	6	6.6		326	206	63.2	
40–59	610	144	10	6.9		466	285	61.2	
≥ 60	263	72	2	2.8		191	97	50.8	
**HBV related departments**					< 0.001				< 0.001
Yes	819	120	17	14.2		699	530	75.8	
No	601	232	6	2.6		369	118	32.0	
**Primary discharge diagnosis**					< 0.001				< 0.001
Yes	512	62	18	29.0		450	438	97.3	
No	908	290	5	1.7		618	210	34.0	
**Total**	**1420**	**352**	**23**	**6.5**		**1068**	**648**	**60.7**	

*Revised case-classification based on information abstracted from patients’ medical record

^#^ Fisher’s exact tests

When we combined all correctly classified and misclassified case-reports in our crude analysis, province, hospital grade, ward type, female, and older patients were statistically associated with having an incorrect case-classification (all p-values < 0.05; Table 3). In the multivariable analysis, misclassified case-reports were more likely to be from Gansu (aOR = 12.7, 95% *CI*:7.7–20.1) and Hainan (aOR = 1.8, 95% *CI*:1.3–2.4), admitted to a grade 2 hospital (aOR = 1.6, 95% *CI*:1.2–2.2), and from a non-HBV-related wards (aOR = 5.3, 95% *CI*:4.1–7.0). Neither age nor gender were statistically significant in the multivariable model.

**Table 3:** Associations between hospital and demographic factors and Hepatitis B case classification to the National Notifiable Disease Reporting System, six project hospitals in Fujian, Hainan, and Gansu Provinces, China, 2015

	Total case-reports	Misclassified Cases	Correctly classified cases	Crude analysis for misclassification		Multivariable analysis for misclassification	
	n	n (%)	n (%)	OR (95%CI)	*p*-value	aOR (95% CI)	*p*-value
**Province**							
Fujian	881	342 (45.7%)	539 (80.3%)	referent	< 0.05	reference	< 0.05
Hainan	267	157 (21.0%)	110 (16.4%)	2.4 (1.8–3.2)		1.8 (1.3–2.4)	
Gansu	272	250 (33.4%)	22 (3.3%)	18.8 (11.8–30.0)		12.7 (7.7–20.1)	
**Hospital Grade**							
2	406	279 (37.2%)	127 (18.9%)	2.6 (2.0–3.3)	< 0.05	1.6 (1.2–2.2)	< 0.05
3	1014	470 (62.8%)	544 (81.1%)	reference		reference	
**Gender**							
Male	991	479 (64.0%)	512 (76.3%)	reference	< 0.05	reference	0.06
Female	429	270 (36.0%)	159 (23.7%)	1.8 (1.5–2.3)		1.3 (0.9–1.7)	
**Age group (years)**							
0–24	130	65 (8.7%)	65 (9.7%)	0.6 (0.4–0.9)	< 0.05	0.6 (0.4–1.1)	0.29
25–39	417	205 (27.4%)	212 (31.6%)	0.6 (0.4–0.8)		0.8 (0.5–1.2)	
40–59	610	315 (42.1%)	295 (44.0%)	0.6 (0.5–0.9)		0.7 (0.5–1.1)	
≥ 60	263	164 (21.9%)	99 (14.8%)	reference		reference	
**HBV related departments**							
Yes	819	272 (36.3%)	547 (81.5%)	reference	< 0.05	reference	< 0.05
No	601	477 (63.7%)	124 (18.5%)	7.8 (6.1–9.9)		5.3 (4.1–7.0)	
**Total**	1420	839 (100%)	671 (100%)	–	–	–	–

The major reason identified for incorrectly reporting patients as having an acute (*n* = 120, 36.5%) or chronic (*n* = 207, 49.3%) infection was lack of additional history or diagnostic testing accompanying abnormal ALT results. The lack of testing following identification of abnormal signs and symptoms as well as ALT test results were also related to incorrect acute (*n* = 36, 10.9%) and chronic reporting (*n* = 68, 16.2%). Additionally, 41 (12.5%) of the misclassified acute HBV infections and 15 (3.6%) of misclassified chronic HBV infections had discharge diagnoses that differed from the corresponding NNDRS case classification (Table 4).

**Table 4:** Characteristics of 749 incorrectly classified Hepatitis B case reports from six project hospitals in Fujian, Hainan, and Gansu Provinces, China, 2015

Characteristics		Numbers of cases	Percentage (%)
**Acute hepatitis case- report (*n*** **= 329)**	ALT abnormal, no other information	120	36.5
	Ultrasound abnormalities or history of HBV	87	26.4
	No HBV history or clinical/ laboratory abnormalities	45	13.7
	Diagnosed as chronic HBV	41	12.5
	Abnormal signs/ symptoms and ALT only	23	7.0
	Abnormal signs/symptoms only	13	4.0
**Chronic hepatitis case-report (*n*** **= 420)**	ALT abnormal, no other information	207	49.3
	No clinical or diagnostic abnormalities (ultrasound performed)	79	18.8
	No clinical or diagnostic abnormalities (ultrasound not performed)	51	12.1
	Abnormal signs/symptoms and ALT only	51	12.1
	Abnormal signs/symptoms only	17	4.1
	Differs from clinical diagnosis	15	3.6

## Discussion

Our evaluation identified discrepancies in the classification of HBV cases reported to NNDRS—only 60% of chronic HBV cases and 7% of acute HBV cases were reported correctly to NNDRS. Most misclassifications involved patients with chronic HBV or HBV carriers or patients without the required diagnostic information who were reported as having an acute infection. Misclassification varied by province, hospital grade, and ward type. Additionally, only a few patients received anti-HBc IgM laboratory tests for differentiating acute and chronic infections as recommended. These discrepancies can be addressed through training, possible revisions to reporting procedures, and development of feedback mechanisms to hospital staff. Our project highlights the importance of routine surveillance evaluations for assessing data quality and monitoring program effectiveness.

Correct case classification in our study was substantially lower than observed in previous evaluations in other locations in China, including in Chongqing, Shandong, Hunan, and Zhejiang Provinces [23–26]. These evaluations suggested that between 63 and 84% of all diagnosed HBV patients were correctly reported to NNDRS. This difference could reflect inconsistency in hepatitis B reporting practices across provinces [27] as well as variability in the methodologies applied in these surveillance evaluations. The WHO has recently prioritized standardized indicator-based surveillance and monitoring tools in the global viral hepatitis elimination strategy that could be used to ensure comparability of the results [2]. Despite these differences, the correct HBV case classification was consistency lower in grade 2 hospitals for all evaluations conducted in China. Because health care staff at grade 3 hospitals in China typically have advanced degrees in medicine and research, these findings could suggest differences in training and clinical awareness. Lack of awareness among health care workers and laboratory staff was also identified as a major gap in viral hepatitis case detection and reporting in a survey on HBV and HCV testing and service delivery in low and middle-income countries [28]. Staffing constraints and doctor-to-patient ratios could also affect the correct case classification and reporting [29, 30]. Targeting lower-level hospital staff with specific interventions, including site visits and regular reviews, may increase the reporting accuracy.

The accuracy of HBV case-reporting also varied by hospital ward. In Fujian, for example, nearly 70% of HBV cases were reported by a ward that was responsible for liver diseases. These patients were primarily seen for liver-related symptoms, resulting in more extensive use of diagnostic testing and facilitating correct case reporting. Most hepatitis B patients (88%) reported by non-liver disease wards—including surgery, pediatric, and gynecologic and obstetrics wards—at the two hospitals in Gansu Province may explain the number of patients who were misclassified in NNDRS. Hospitalized patients in China are routinely tested for HBV infection, and in order to help prevent mother-to-child transmission (PMTCT), the NHC implemented a national policy requiring antenatal HBsAg testing for pregnant women in 2010 [31]. These strategies have increased HBV testing in non-liver disease wards. Strengthening the training on HBV case definitions for clinicians, especially for non-liver disease wards, could greatly improve the accuracy of HBV diagnosis.

Regardless of the ward, laboratory testing necessary for differential diagnosis was limited for the HBV patients reported by the six participating hospitals. Only one-third of the reported acute HBV patients received testing for hepatitis A infections, and only 40 and 1.2% of patients seen in the liver disease wards were tested for anti-HEV IgM and anti-HBc IgM respectively. Frequency of testing for anti-HEV IgM and anti-Hbc IgM was even lower in non-liver disease wards. Because anti-HBc IgM titers can be used to differentiate acute and chronic infections, WHO recommends anti-HBc IgM testing to assist with diagnosing recent infections, particularly if the patient’s medical history or previous testing results are unknown. In our project, HBV patients were commonly misclassified as acute because of a lack of previous medical history and laboratory testing. Most of these patients would have likely been correctly classified as having chronic HBV by anti-HBc IgM testing if it had been performed as recommended [14]. Insufficient testing due to lack of clinical awareness or cost of testing was identified as a gap in generating quality epidemiologic data on hepatitis B infections in high burden countries [32].

Our findings may also reflect the current NNDRS reporting procedures. Testing hospitalized patients in China for HBsAg is routine practice [33], and all clinicians are required to report hepatitis B cases to NNDRS within 24 h of the initial diagnosis. Although clinicians can revise NNDRS case reports after additional diagnostic information becomes available, there is currently no incentive to do so. These classification discrepancies could be minimized by training hospital staff to reclassify NNDRS case reports when the final discharge diagnosis has been recorded in the hospital information system. Additionally, mechanisms should be created to ensure that regular reviews (i.e., monthly) are conducted to assess classification of NNDRS HBV case-reports. Routine feedback from China CDC on findings from the national hepatitis B surveillance program could also help local clinicians improve patient diagnosis and reporting practices [34].

Surveillance data are used to generate regional, national, and global estimates of acute and chronic HBV infections. The misclassification errors identified in our evaluation, as well as in previous studies, suggest that the incidence and prevalence of acute HBV and chronic HBV calculated from NNDRS are likely inaccurate. If we apply our re-classification results to the national HBV case-data from 2015, for example, the number of acute infections may be overestimated by as much as 37,000 and the number of chronic infections could be underestimated by more than 300,000. These inaccuracies result in major challenges in identifying HBV outbreaks, evaluating the effectiveness of current prevention measures, and in efficiently allocating health care resources [19]. The 17 correctly classified acute infections from the two hospitals in Hainan, for example, could suggest missed opportunities to vaccinate and should be further investigated.

Our evaluation had several limitations. First, we conducted the evaluation in six hospitals in three provinces, limiting the generalizability of the results. Because of differences in the service area and population size of the project hospitals, our results do not reflect the incidence or prevalence of hepatitis B in the local population. Second, we targeted grade 2 and 3 hospitals; surveillance quality in lower level healthcare facilities was not evaluated. Based on our findings, additional training and support for HBV case-reporting at these lower level facilities would likely also be beneficial. Third, almost 30% of HBV case-reports to NNDRS could not be classified because of missing clinical information or testing results in LIS. We were also unable to evaluate reporting classification of outpatients who did not receive a hospital record number. The accuracy of the case classification of these HBV case-reports currently remains unknown. New approaches are needed to ensure that all HBV patients are identified and correctly reported to NNDRS. Finally, we were not able to assess the extent of under-reporting, meaning those patients who were diagnosed with acute or chronic HBV infections but who were not reported to NNDRS.

## Conclusion

Our surveillance evaluation at six hospitals in three provinces identified discrepancies in the classification of HBV case-reports to NNDRS. Onsite training on the use of anti-HBc IgM testing as well as on HBV case definitions and reporting procedures are needed to accurately assess program effectiveness and ensure that patients are referred for the appropriate treatment and care. Routine feedback mechanisms from the national hepatitis B surveillance program would also be beneficial. Because surveillance data are used to monitor program effectiveness and progress toward global viral hepatitis elimination goals, our findings support the use of similar evaluations in other locations as a possible complement to resource-intensive HBV sero-surveys.

## Data Availability

The datasets generated and/or analyzed during the current study are not publicly available due to reasons of patient confidentiality but are available from the corresponding author on reasonable request.

## Abbreviations

HBV: Hepatitis B virus
WHO: World Health Organization
HBsAg+: HBV surface antigen positive
NHC: National Health Commission
NNDRS: National Notifiable Disease Reporting System
LIS: Laboratory information system
HIS: Hospital information system
China CDC: Chinese Center for Disease Control and Prevention
anti-HBc IgM: HBV core antibody IgM
anti-HAV IgM: Antibody IgM for hepatitis A virus
anti-HCV: Antibody for hepatitis C virus
CT: Computerized tomography
MRI: Magnetic Resonance Imaging
HCC: Hepatocellular carcinoma
OR: Odds ratios
CI: Confidence intervals
