# Correction to: Using a hepatitis B surveillance system evaluation in Fujian, Hainan, and Gansu provinces to improve data quality and assess program effectiveness, China, 2015

**DOI:** 10.1186/s12879-020-05708-x

**Published:** 2020-12-21

**Authors:** Hui Zheng, Alexander J. Millman, Jeanette J. Rainey, Fuzhen Wang, Rui Zhang, Hong Chen, Zundong Yin, Huaqing Wang, Guomin Zhang

**Affiliations:** 1 National Immunization Program, Chinese Center for Disease Control and Prevention, Beijing, China; 2 Division of Viral Hepatitis, United States Centers for Disease Control and Prevention, Atlanta, GA USA; 3 Division of Global Health Protection, United States, Centers for Disease Control and Prevention, Atlanta, GA USA; 4 Emerging Infections Program, Chinese Center of Disease Control and Prevention, Beijing, China

**Correction to: BMC Infect Dis 20, 547 (2020)**

**https://doi.org/10.1186/s12879-020-05265-3**

After publication of the original article [1], the authors identified an error in Fig. 1.

The correct Fig. 1 is given below:

**Fig. 1:**
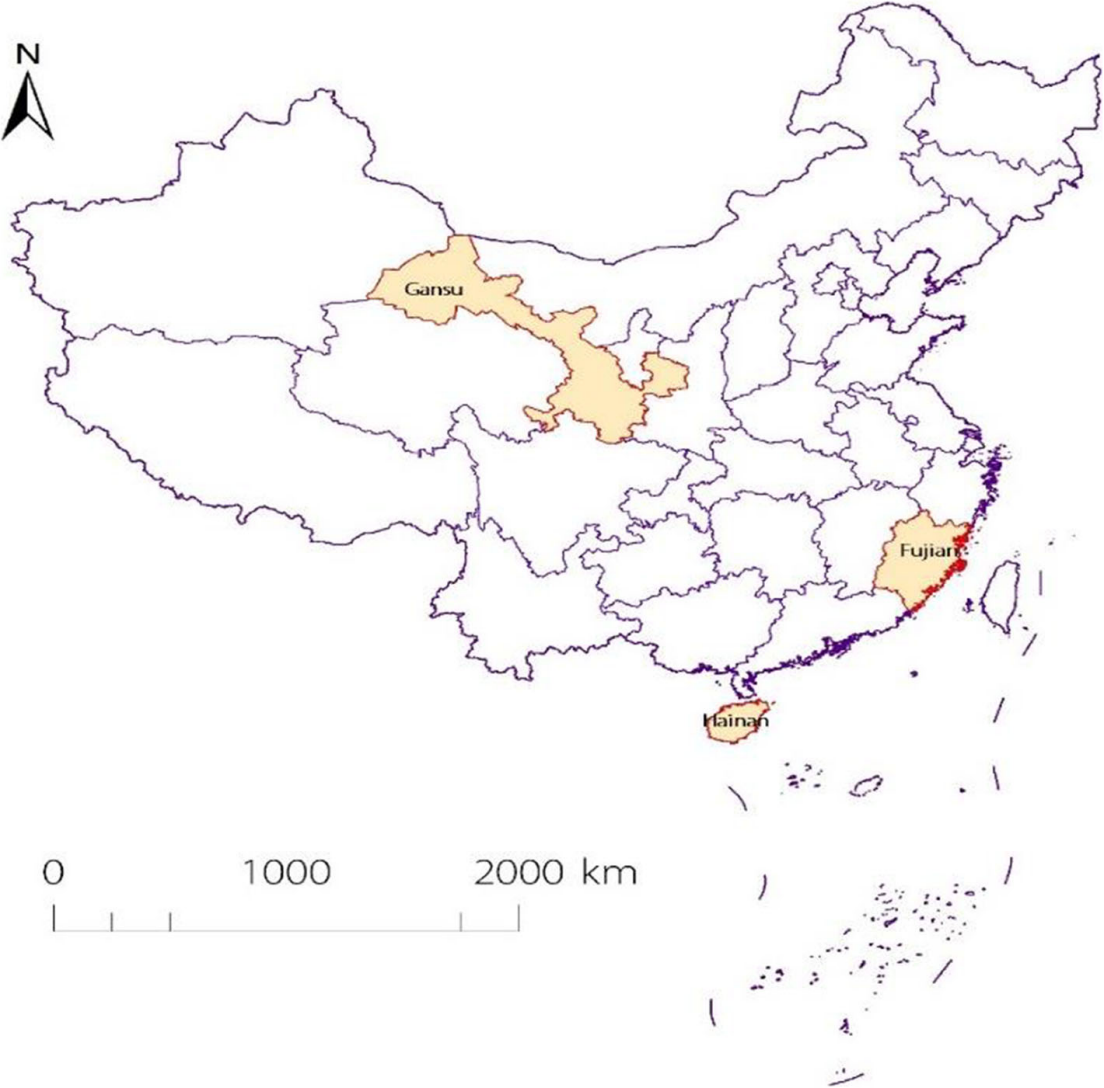
Location of Fujian, Hainan, and Gansu Provinces participating in the hepatitis B surveillance project, China 2015
